# The Very High Premature Mortality Rate among Active Professional Wrestlers Is Primarily Due to Cardiovascular Disease

**DOI:** 10.1371/journal.pone.0109945

**Published:** 2014-11-05

**Authors:** Christopher W. Herman, Anna S. C. Conlon, Melvyn Rubenfire, Andrew R. Burghardt, Stephen J. McGregor

**Affiliations:** 1 Eastern Michigan University, Ypsilanti, Michigan, United States of America; 2 University of Michigan, Ann Arbor, Michigan, United States of America; University of Bologna, Italy

## Abstract

**Purpose:**

Recently, much media attention has been given to the premature deaths in professional wrestlers. Since no formal studies exist that have statistically examined the probability of premature mortality in professional wrestlers, we determined survival estimates for active wresters over the past quarter century to establish the factors contributing to the premature mortality of these individuals.

**Methods:**

Data including cause of death was obtained from public records and wrestling publications in wrestlers who were active between January 1, 1985 and December 31, 2011. 557 males were considered consistently active wrestlers during this time period. 2007 published mortality rates from the Center for Disease Control were used to compare the general population to the wrestlers by age, BMI, time period, and cause of death. Survival estimates and Cox hazard regression models were fit to determine incident premature deaths and factors associated with lower survival. Cumulative incidence function (CIF) estimates given years wrestled was obtained using a competing risks model for cause of death.

**Results:**

The mortality for all wrestlers over the 26-year study period was.007 deaths/total person-years or 708 per 100,000 per year, and 16% of deaths occurred below age 50 years. Among wrestlers, the leading cause of deaths based on CIF was cardiovascular-related (38%). For cardiovascular-related deaths, drug overdose-related deaths and cancer deaths, wrestler mortality rates were respectively 15.1, 122.7 and 6.4 times greater than those of males in the general population. Survival estimates from hazard models indicated that BMI is significantly associated with the hazard of death from total time wrestling (p<0.0001).

**Conclusion:**

Professional wrestlers are more likely to die prematurely from cardiovascular disease compared to the general population and morbidly obese wrestlers are especially at risk. Results from this study may be useful for professional wrestlers, as well as wellness policy and medical care implementation.

## Introduction

Much attention has been given in the popular media to the occurrence of premature deaths associated with professional wrestling, a choreographed form of high-impact athletic entertainment. Over the past twenty-five years, professional wrestling has become increasingly popular within the United States and numerous national wrestling organizations were in existence throughout the 1980s, 1990s, and early 2000s. World Wrestling Entertainment Inc. (WWE) is currently the largest professional wrestling organization in the world, generating nearly $500 million annually in revenues [1].

The potential for both acute and chronic injury exists among professional wrestlers, especially when considering the intensity of their efforts and their year-round competition. Abuse of androgenic anabolic steroids (AAS) was especially prevalent in professional wrestling throughout the 1980s and early 1990s until a widely publicized federal trial regarding AAS abuse in 1992 [2]. Both AAS and analgesic abuse have been linked to cardiovascular disease [3], [4]. The abuse of these drugs may play a role in the premature cardiac-related deaths of professional wrestlers that receive media attention.

While most professional wrestlers have an exaggerated muscular physique, many are visibly obese with truncal obesity suggesting the presence of the metabolic syndrome (pre-diabetes) and some are morbidly obese [5], [6]. There is a consistent relationship between obesity defined by the body mass index (BMI), visceral fat represented by waist, diabetes, hypertension, coronary disease, stroke, as well as premature death [7]–[9]. While it is understood that BMI is not the most effective measurement of body fatness among a highly muscular athletic population such as professional wrestlers, BMI values may be more representative of the actual body compositions of the wrestlers in the higher BMI ranges. In a recent study of NFL players, increased size measured by BMI was associated with increased blood pressure (BP), as well as increased triglycerides, LDL cholesterol, and fasting glucose levels, as well as lower HDL cholesterol levels [10].

The media has closely examined professional wrestler deaths that may be associated with some of the aforementioned occupational and lifestyle factors, but no epidemiological research has formally analyzed the mortality rates and survivability of the wrestlers over a long period of time. Therefore, the purpose of this study was to determine the survival estimates for professional wrestlers active between 1985 and 2011. Mortality of professional wrestlers was compared to that of the general population.

## Methods

### Ethics Statement

All data were de-identified for the purposes of this analysis.

A comprehensive review was conducted of the 557 regularly employed male professional wrestlers who were consistently active between January 1, 1985 and December 31, 2011. All subjects were employed by various national professional wrestling organizations during this time period [11]–[14]. Data was collected between January 2009 and December 2011. No permits were required for the described study, which complied with all relevant regulations. Active wrestlers who made their professional debut after June 30, 2011 were excluded. Regular employment was defined as performers featured on pay-per-view television broadcasts, live television programming, television tapings, or those who traveled as a featured performer on untelevised arena tours (also known as house shows) for at least six months or longer. Wrestler birth name, stage name, billed height, billed weight, BMI, date of birth, sex, race, age, and when applicable, cause of death, date of death, and age at death were collected from various public sources including The Wrestling Observer Newsletter [11], other wrestling-related internet sites [13], [15]–[17], and the WWE's officially licensed published Encyclopedia [14]. Complete information was available for 536 of the 557 wrestlers who were entered into survival analyses and hazard models described below.

The effects of age, BMI, height, weight, and the amount of time wrestled on five year and ten year survival were assessed using logistic regression models. Definitions for body mass index (kg/m^2^) were as follows: optimal 20–24.9; overweight 25–29.9; stage 1 obesity 30–34.9; stage 2 obesity 35–39.9; and stage 3 or morbid obesity equal to or greater than 40 kg/m^2^.

### Calculations of Mortality Rates

Mortality rates in the professional wrestlers were calculated and expressed as a percentage over time in years and as deaths per 100,000 per year and determined by age and cause of death. The rates were compared to the most recently available published population rates from the Centers for Disease Control (CDC) in 2007 [18]. The total number of person-years for each age cohort was calculated, and the expected number of deaths for each age group was obtained based on the 2007 CDC reported mortality rates for each group. The standardized mortality ratio (SMR) was calculated as the observed number of deaths divided by the expected number of deaths for each age group. Finally, P-values were calculated using a chi-square test to determine whether the observed number of deaths in each age group was significantly different from the expected number of deaths, with the null hypothesis being that the death rate of wrestlers within each age group is equal to the United States population death rate for that group.

### Survival Analysis

Kaplan-Meier survival estimates were obtained and Cox proportional hazards models were fit to determine incident professional wrestlers' premature death and factors associated with lower survival rates. Logistic regression models were used to determine factors related to the odds of five year and ten year survival after start of career. The Kaplan-Meier plot for years wrestled at time of death does not take into account years wrestled prior to 1985. In a separate Cox regression analysis we found the time wrestled prior to 1985 was not significantly associated with the hazard of death for years wrestled after 1985. Therefore, Kaplan-Meier analyses and hazard models only consider survival probability from the start of a wrestler's career (or 1985, whichever was later). Product-limit survival estimates were also determined for wrestlers by cause of death.

Statistical analysis was performed using the R statistical software package version 2.13.1 and SAS statistical software, version 9.2 [19], [20].

## Results

557 males (495 survivors and 62 deceased) were included in this study. All survivor ages were calculated as of December 31, 2011. The average age when subjects started their professional wrestling career was 29.0±6.4 years. Approximately 78% of the wrestlers in this study were Caucasian, while Hispanic, Black, Asian/Pacific Islander and wrestlers of other races consisted of approximately 9%, 6%, 6%, and 1% of the sample, respectively. Demographic data for survivors and wrestlers who died within five years and ten years from the start of their career are displayed in Table 1 and Table 2, respectively.

**Table 1:** Five-Year Survival for Professional Wrestlers.

Variable	Total (n = 557) Mean (SD)	Alive 5 years after start (n = 503 out of 515 with at least 5 years of follow-up) Mean (SD)	Dead by 5 years after start (n = 12 out of 515 with at least 5 years of follow-up) Mean (SD)	P-value for odds of death by 5 years, given start age
Starting Age (yrs)	29.3 (6.4)	29.3 (6.6)	32.0 (6.6)	0.17
BMI (kg/m^2^)	33.3 (5.1)	33.4 (4.9)	37.7 (10.7)	0.01*
Height (m)	1.87 (0.13)	1.87 (0.09)	1.87 (0.13)	0.90
Weight (kg)	116.8 (24.9)	117.1 (24.1)	135.5 (58.5)	0.03*

* Statistical significance between survivors and deceased at the 0.05 level

**Table 2:** Ten-Year Survival for Professional Wrestlers.

Variable	Total (n = 557) Mean (SD)	Alive 10 years after start (n = 419 out of 445 with at least 10 years of follow-up) Mean (SD)	Dead by 10 years after start (n = 26 out of 445 with at least 10 years of follow-up) Mean (SD)	P-value for odds of death by 10 years, given start age
Starting Age (yrs)	29.3 (6.4)	29.7 (6.7)	31.2 (7.1)	0.28
BMI (kg/m^2^)	33.3 (5.1)	33.5 (5.0)	37.0 (8.2)	0.004*
Height (m)	1.9 (0.1)	1.86 (0.09)	1.90 (0.14)	0.07
Weight (kg)	116.8 (24.9)	117.2 (23.6)	136.2 (48.8)	0.001*

* Statistical significance between survivors and deceased at the 0.05 level

All wrestler mortality rates were compared to those of 2007 CDC published mortality rates [18]. The total mortality over the 26-year study period was 0.007 per person-year, or 708 deaths per 100,000 per year. Specific mortality rates by BMI category for wrestlers are illustrated in Table 3. A comparison between wrestler age-adjusted mortality rates and CDC mortality rates for the general population are detailed in Table 4.

**Table 3:** Comparison of Mortality rates by BMI Categories for Wrestlers by Gender.

BMI Category by # of males	Mortality rate Per 100,000 (Males)	Hazard Ratio (Vs. Optimal Group)
Optimal (n = 5)	0	1.00
Overweight (n = 106)	36	1.98
Obese 1 (n = 326)	5197	3.91
Obese 2 (n = 81)	3047	7.73
Morbidly Obese (n = 37)	6810	15.29

**Table 4:** Comparison of Total Wrestler and CDC Mortality rates by Age Group.

Age Group (years)	Person- years in group	# Dead in Age Group	CDC Mortality Rate (Per 100,000)	Expected deaths (person-years *CDC rate/10000)	SMR (observed deaths/expected)	Chi-square p-value
20–24	252	0	145	0.37	0	0.82
25–29	1118	2	140	1.57	1.28	0.96
30–34	1697	13	148	2.51	5.18	<0.0001*
35–39	1698	9	185	3.14	2.87	0.002*
40–44	1395	12	277	3.86	3.11	0.0001*
45–49	1077	13	424	4.57	2.85	0.0002*
50–54	713	3	647	4.61	0.65	0.60
55–59	421	2	922	3.88	0.52	0.48
60–64	236	4	1328	3.13	1.28	0.84
65–69	109	3	2002	2.18	1.37	0.83
70–74	23	0	3047	0.70	0	0.81
75–79	8	1	4817	0.39	2.59	0.85

*Statistical significance between wrestler and 2007 CDC rates at the 0.05 level.

Overall, wrestler mortality rates for age groups 25–34, 35–44, and 45–54 were 1.3, 2.9, and 2.9 times greater than the 2007 CDC mortality rates for males of the same age groups. Mortality rates were also compared between the wrestlers and general population for a variety of causes of death. Among the wrestler deaths, the percentages of wrestler deaths attributed to cardiovascular disease, acute drug intoxications, and cancer were 35.5%, 17.8%, and 8.8% of total deaths, respectively. For cardiovascular-related deaths, drug overdose-related deaths and cancer deaths, the wrestler mortality rates were respectively 15.1, 122.7 and 6.4 times greater than those of males in the general population [18].

### Survival Estimates

**Figure 1:**
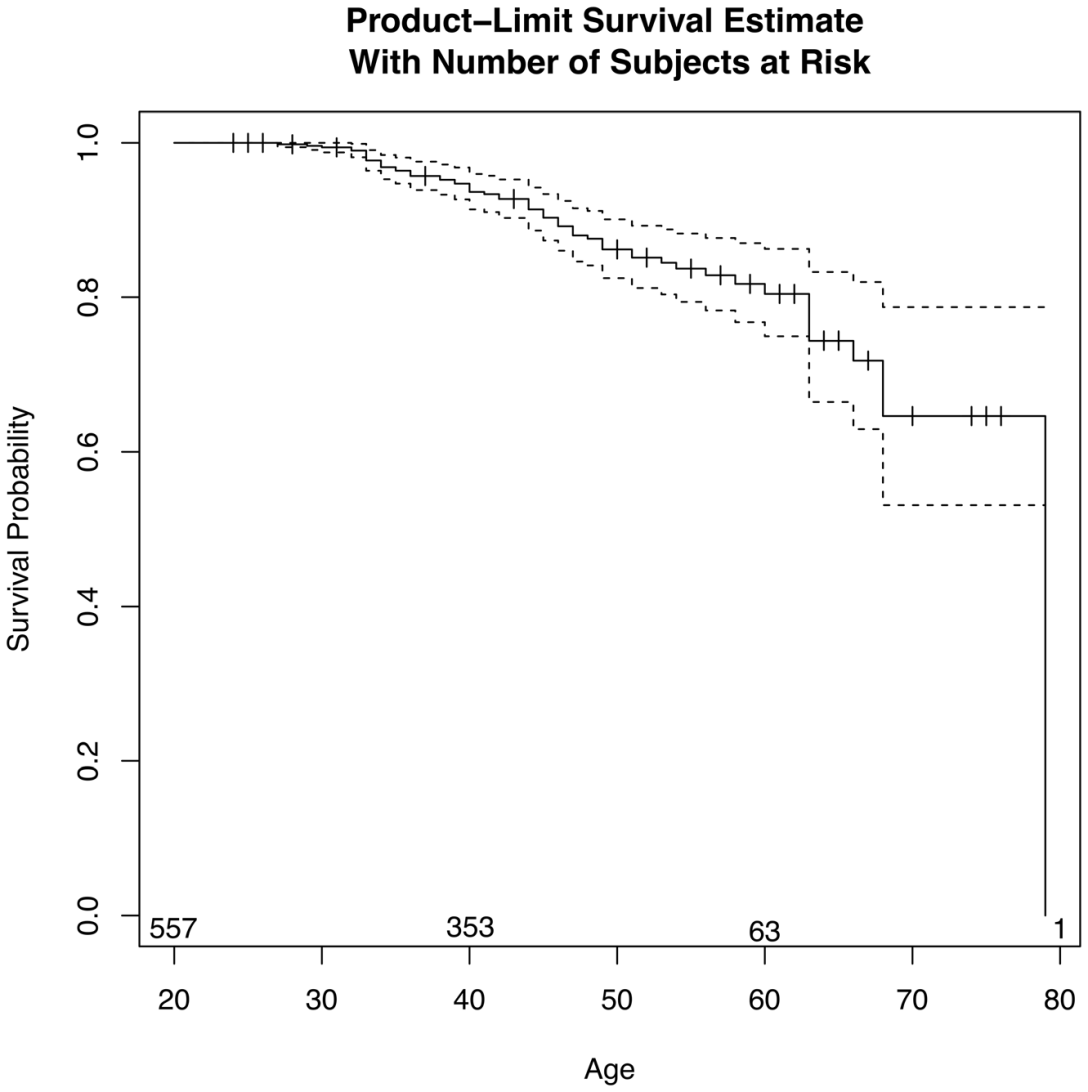
Survival probability by age.

Because the CDC does not publish age-adjusted mortality rates by cause of death, a series of survival analyses were completed to develop a more formal understanding of the survivability of professional wrestlers. Survival estimates and 95% confidence intervals (CI) are presented in Figure 1. Approximately 16% of wrestlers died by the time they were 50 years of age. Additionally, survival estimates and 95% CI from the start of the wrestler's career or 1985 (whichever is later) were calculated. A professional wrestler who has been active for 10 years has a 5% mortality (survival  = 0.95 (95% CI: 0.93, 0.97)), compared with an 18% mortality (survival  = 0.82 (95% CI: 0.77, 0.87)) 25 years after the start of their career. The wrestler's starting age was not significantly associated with the hazard of mortality over time after starting their wrestling career (HR 1.03 (95% CI: 0.99, 1.06); p = 0.13) with every increase in starting age category.

**Figure 2:**
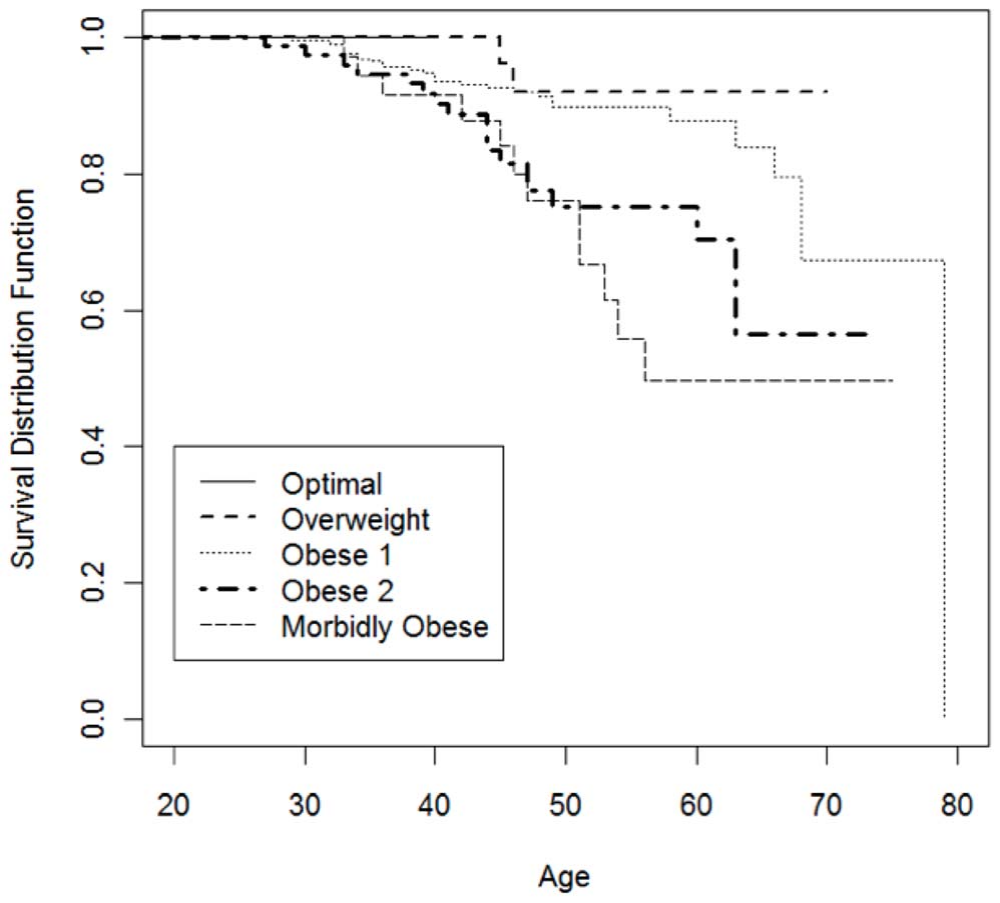
Survival probability for BMI by age for wrestlers.

**Figure 3:**
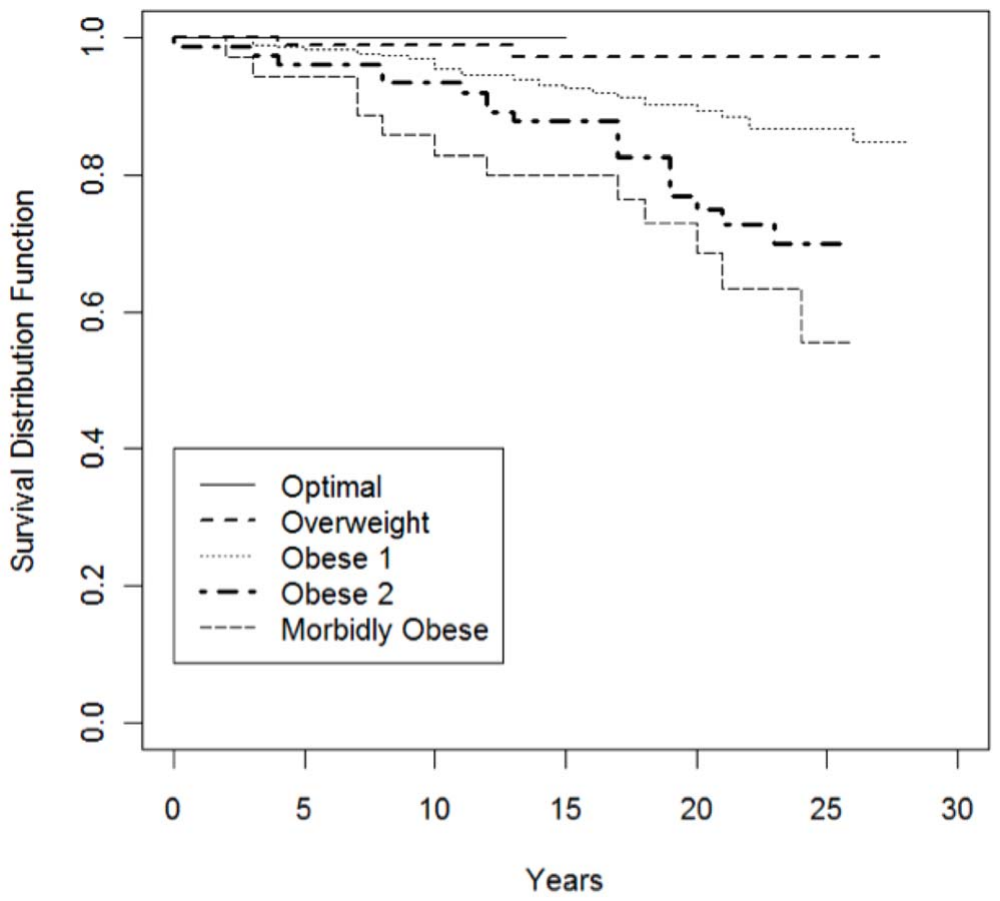
Wrestler survival probability for BMI by years from start of career.

**Figure 4:**
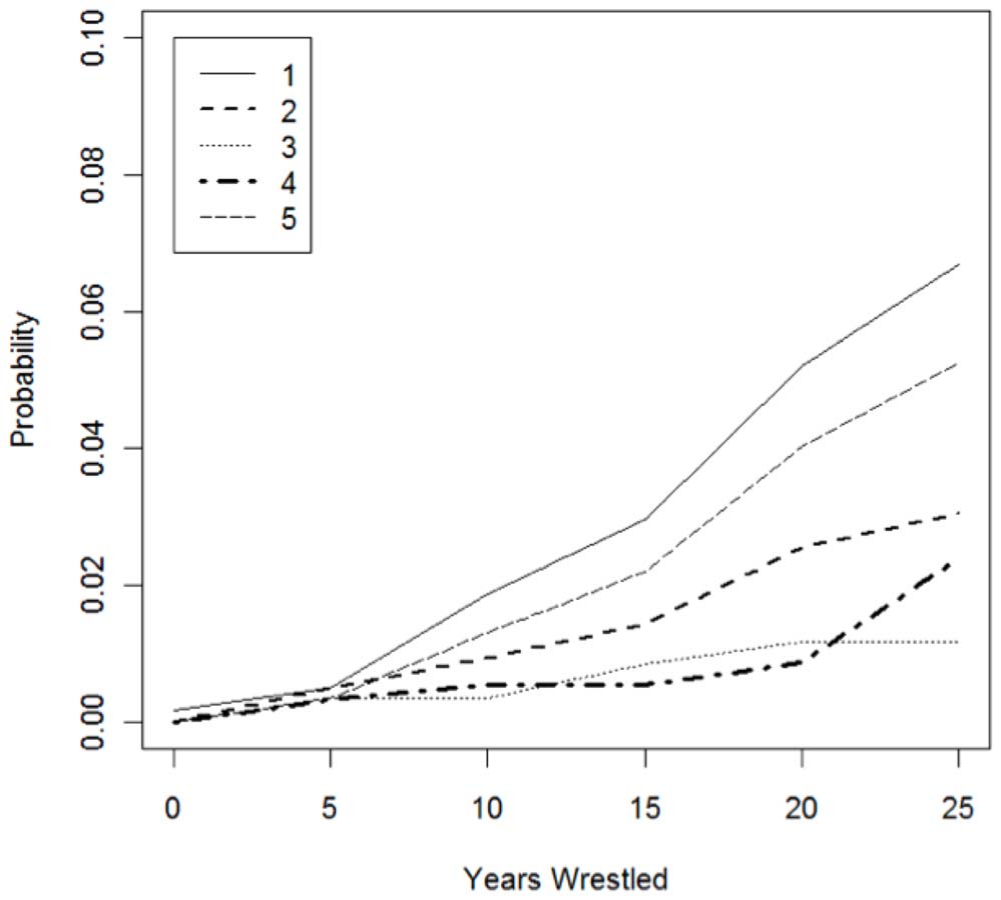
Cumulative incidence function estimates given years wrestled for cause of death. Figure 4 Note: 1 =  Cardiovascular-Related Death; 2 =  Drug-Related Death; 3 =  Non-Drug Suicide and Homicide; 4 =  Cancer Death; 5 =  Other Cause of Death.

Figure 2 displays survival probability by BMI category by age for the wrestlers. In a univariate Cox proportional hazards model for survival, BMI was significantly associated with overall survival, with the hazard ratio for death increasing by a factor of 1.86 (95% CI: 1.40, 2.48; p<0.0001) for each increase in BMI category. Figure 3 reports survival probability for BMI by years since the start of a wrestling career. A separate Cox proportional hazards model for survival found that BMI was significantly associated with the hazard of death after controlling for age. The hazard ratio (HR) for death increased by a factor of 2.12 (95% CI: 1.50, 2.81; p<0.0001) for each increase in BMI category. Figure 4 demonstrates the probability of death by causes (e.g. cardiovascular disease, drug-related death) given the number of years wrestled.

## Discussion

Our study of active professional wrestlers over the past quarter of a century confirms there is an alarmingly high premature mortality rate. Wrestlers between the ages of 25 and 49 were 4.5 times more likely to die than the general population as reported by the CDC in 2007. Leading causes of mortality among wrestlers, as calculated using cumulative incidence functions, were cardiovascular-related (22 out of 62 deaths; 38%), drug-related (including overdoses of prescription drugs; 14.8%), and cancer (11.5%) throughout a 26-year wrestling career. Professional wrestlers who survived to their mid-fifties had a subsequent mortality rate comparable to the general population, as reported in Table 3.

Ninety-eight percent of wrestlers had a BMI ranging from overweight to morbidly obese, and approximately 21% were classified as stage 2 or morbidly obese. The overall mean [SD] BMI for the surviving wrestlers in this study was 32.7 kg/m2 [±4.4] and mean BMI for deceased wrestlers was 37.1 [±8.1] by last available weight. A similar association between obesity in professional athletes and mortality has been demonstrated in retired National Football League (NFL) Players. In a report from 1994, retired NFL players experienced an overall 46% lower mortality rate compared with the general population, but retired offensive and defensive linemen with a higher BMI had a 52% greater risk of dying from heart disease [21].

In the current study, obesity as defined by categories of BMI was associated with total mortality for wrestlers. Morbidly obese wrestlers had a 14% chance of mortality after only seven years of competition, and this increased to 23% after twenty years of wrestling. Duration of active wrestling has a strong relationship with mortality. Those who debuted prior to age 25 years had a 16% chance of mortality after twenty years of competition. Specifically, the aforementioned cumulative incidence function estimates indicated that there was a higher incidence of death among wrestlers from drug-related and cardiovascular causes over the 26-year study period.

Our findings suggest many of the premature deaths in active professional wrestlers result from adverse health behaviors including the abuse of performance enhancing drugs, the abuse of prescription and non-prescription drugs, and obesity that may be related to the duties performed and injuries sustained [3], [4], [6], [22]–[24]. It is reasonable to assume chronic musculoskeletal injuries resulting in abuse of pain-relieving prescription drugs play a role in the premature drug-related deaths and possibly suicide among some wrestlers. It is also plausible that the painful injuries are self-treated with high doses of non-steroidal anti-inflammatory drugs (NSAIDs), which can cause or worsen hypertension and increase the risk of heart and kidney failure [25]–[27].

Considering the increasing popularity of professional wrestling as a form of entertainment, it would seem prudent for the wrestlers and their employers to further establish health screening efforts and treatments designed to prevent premature deaths related to performance enhancing drugs, prescription drugs, NSAIDs, eating disorders, and metabolic (diabetes) and cardiovascular diseases. Based upon our findings and the known association between obesity and hypertension, professional wrestlers should be regularly advised regarding their blood pressure and risk factors for hypertension, and treated appropriately. Even mild increases in blood pressure are associated with an increase in cardiovascular event (CVE) rate. There is a direct, linear relationship between BP and risk of stroke and other cardiovascular events with a systolic BP of greater than 115 mmHg; a 20 mmHg increment of systolic BP doubles the risk of CVE across a range from 115/75 to 185/115 mmHg [28]. Subjects with high normal BP are at twice the risk of developing hypertension than those with lower values and therefore over time are at greater risk of events and may require antihypertensive medication in the future.

To our knowledge, there is no published reference regarding blood pressure and prevalence of hypertension in professional wrestlers. However, there have been studies of active professional football players including offensive and defensive linemen who have a similar body habitus, similar training, potential for anabolic steroid abuse, and performance associated injuries requiring pain relief. Compared to over 1900 healthy matched controls, among 504 NFL professional football players with an average age of 26 years, there was a nearly 3 fold (13.8%% vs. 5.5%) incidence of hypertension (BP>140/90 mmHg) and prehypertension (120–139 mmHg systolic or 80–89 mmHg diastolic) (64.5% vs. 24.2%), p<0.001 for both. The mean BMI of offensive and defensive lineman was 37.8 kg/m^2^ and 35.7 kg/m^2^ respectively. The combined prevalence of hypertension and prehypertension was 91% in the largest players. Higher BMI was associated with each of the measurable coronary risk factors including higher BP, LDL cholesterol, triglycerides, and fasting glucose, and decrease in HDL cholesterol [10]. The NFL has plans to investigate the mechanisms of hypertension including the use of NSAIDs, strength and resistance training, salt intake, and sleep disordered breathing.

It is well known that diabetes mellitus (DM) is a metabolic disorder characterized by increased mortality rates and importantly implicated in the atherogenic process [29]. Further, in addition to vascular disease, diabetes is associated with substantial premature death from several cancers, infectious diseases, external causes, intentional self-harm, and degenerative disorders, independent of several major risk factors [30]. While we don't have data on the prevalence of diabetes in our wrestler cohort, considering their BMI [10] and use of steroids, and androgens, it is highly likely there is an increase incidence of both diabetes and the metabolic syndrome whose components are each risk factors or markers for coronary disease. Moderate to severe obesity, both central and abdominal, which is very prevalent in the wrestlers, is associated with an abnormal fasting blood sugar, hypertension, low HDL cholesterol, and elevated triglycerides, each of which increases the risk of diabetes and coronary atherosclerosis, coronary events, and cardiovascular mortality [31].

It is important to note that many of the professional wrestlers analyzed in the present study did not directly compete for WWE, which is currently the world's largest professional wrestling organization that instituted a Talent Wellness Program in 2006 to address some of the health concerns among their performers [32], [33]. The program components include ‘an aggressive substance abuse and drug testing policy' and ‘a cardiovascular testing and monitoring program’ [32]. Currently, there is one other national professional wrestling organization and information on any associated corporate wellness policy is not publicly available. Results from this study are associated with professional wrestlers in the industry as a whole and not with any specific professional wrestling organization.

There are several limitations to our study, the primary being the reliability of data collection. Rather than the standard of medical record review for each participant, we used non-scientific websites and other public data sources such as publications and websites. These sources of information are similar in nature to those employed by a recent study of professional cyclists [34]. We also used cause-of-death information from these sources to compare mortality rates with the CDC, which more formally confirms causes of death. The veracity of information in the present study is supported by the fact that premature wrestler deaths have been publicized and extensively scrutinized. In conjunction with the publicly acknowledged wellness violations among some of these individuals, the circumstances of these deaths are publicly accepted. We concluded the information obtained is reasonable and can be used to support more accurate studies using standard methods with medical records. Additionally, in this observational study, it is not appropriate to state that a causal relationship exists between participation in a professional wrestling organization and survivability. With the exception of age, height, weight, BMI, and time-period wrestled, we were not able to adjust for any other potential confounding factors. This is somewhat similar to the aforementioned cycling study [34]. Furthermore, while we defined obesity by BMI, which has not been validated in wrestlers with scientific methods of measuring body fatness, it is reasonably assumed that any potential exaggeration in weight per unit height among the wrestlers was likely consistent across all BMI categories for all of the performers. In the present study, while there was no loss to follow-up for overall mortality, the cause and/or date of mortality were not found for a few of the deceased wrestlers and it is possible that a wrestler may have met the requirements for dataset inclusion but was accidentally omitted. Finally, small sample sizes for the wrestlers <25 years and >56 years of age limited the analysis in these groups.

## Conclusion

Considering the very high rate of premature mortality from cardiovascular disease, cancer, and substance abuse, the health and wellbeing of professional wrestlers who are part of the entertainment industry need an intense prevention strategy with aggressive treatment of each of the risk factors. Results from this study may be useful for professional wrestlers, and may be useful for the development of future wellness policy and medical care implementation among professional wrestling organizations.
